# Supplementary material for: Identification of Endometriosis Pathophysiologic-Related Genes Based on Meta-Analysis and Bayesian Approach
Source: Int J Mol Sci. 2025 Jan 6;26(1):424. doi: 10.3390/ijms26010424 (PMC11720405; doi:10.3390/ijms26010424)
Supplement: Supplementary file 1 [file ijms-26-00424-s001.zip › ijms-3366398-supplementary.pdf]

Identification of Endometriosis Pathophysiologic-Related Genes Based on Meta-Analysis and Bayesian Approach

Jieun Kang [1,†], Kwangjin Ahn [2,†], Jiyeon Oh [3], Taesic Lee [4], Sangwon Hwang [5], Young Uh [2,*] and Seong Jin Choi [1,*]

[1] Department of Obstetrics and Gynecology, Yonsei University Wonju College of Medicine, 20, Ilsan-ro, Wonju-si 26426, Republic of Korea; k131730@hanmail.net

[2] Department of Laboratory Medicine, Yonsei University Wonju College of Medicine, 20, Ilsan-ro, Wonju-si 26426, Republic of Korea; kjahn123@yonsei.ac.kr

[3] Department of Global Medical Science, Yonsei University Wonju College of Medicine, 20, Ilsan-ro, Wonju-si 26426, Republic of Korea; jiyeon06@yonsei.ac.kr

[4] Department of Family Medicine, Yonsei University Wonju College of Medicine, 20, Ilsan-ro, Wonju-si 26426, Republic of Korea; ddasic123@yonsei.ac.kr

[5] Department of Precision Medicine, Yonsei University Wonju College of Medicine, 20, Ilsan-ro, Wonju-si 26426, Republic of Korea; arsenal@yonsei.ac.kr

* Correspondence: u931018@yonsei.ac.kr (Y.U.); choisj@yonsei.ac.kr (S.J.C.); Tel.: +82-33-741-1592 (Y.U.); +82-33-741-1278 (S.J.C.)

† These authors contributed equally to this work.

Supplementary data

Figures S1. Cell signaling pathways of differentially expressed genes (DEGs) at GSE6364. 3

Figures S2. Cell signaling pathways of differentially expressed genes (DEGs) at GSE73622-MSC. 5

Figures S3. Cell signaling pathways of differentially expressed genes (DEGs) at GSE73622-SF. 6

Figures S4. Cell signaling pathways of differentially expressed genes (DEGs) at GSE141549-GPL13376 (Bi). 7

Figures S5. Cell signaling pathways of differentially expressed genes (DEGs) at GSE141549-GPL13376 (Or). 8

Figures S6. Cell signaling pathways of differentially expressed genes (DEGs) at GSE141549-GPL10558. 9

Figures S7. Cell signaling pathways of differentially expressed genes (DEGs) at endometriosis presence meta-analysis. 11

Figures S8. Cell signaling pathways of differentially expressed genes (DEGs) at endometriosis presence meta-analysis. 12

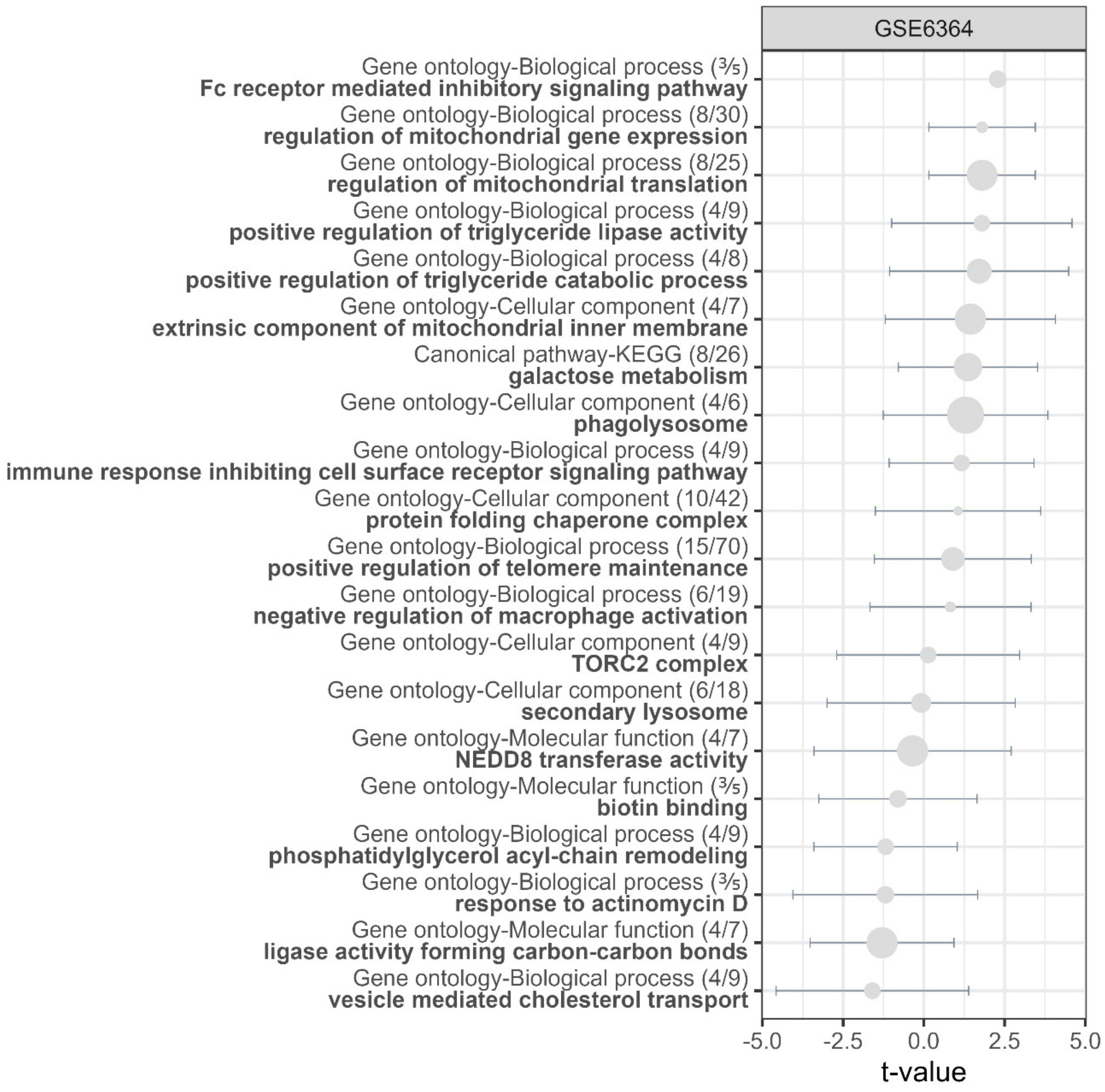

Figures S1. Cell signaling pathways of differentially expressed genes (DEGs) at GSE6364. The analysis identifies the cell signaling pathways associated with DEGs with P below 0.05. The y-axis displays the information about the pathways, with the names of databases containing cell signaling pathways shown at the top. Following this, the total number of genes in each pathway (denoted as the denominator) and the number of DEGs in that pathway (indicated as the numerator) are presented in parentheses. The pathway names are highlighted in bold text. The x-axis represents the t-values of the DEGs within each pathway. Error bars indicate the range of ±1 standard deviation from the mean t-value. Each dot's position corresponds to the

mean t-value, while its size reflects the log-transformed *P* from the pathway analysis. The color of the dot represents the significance level of *P*, with gray indicating values equal to or above 0.05. There are no significant pathways.

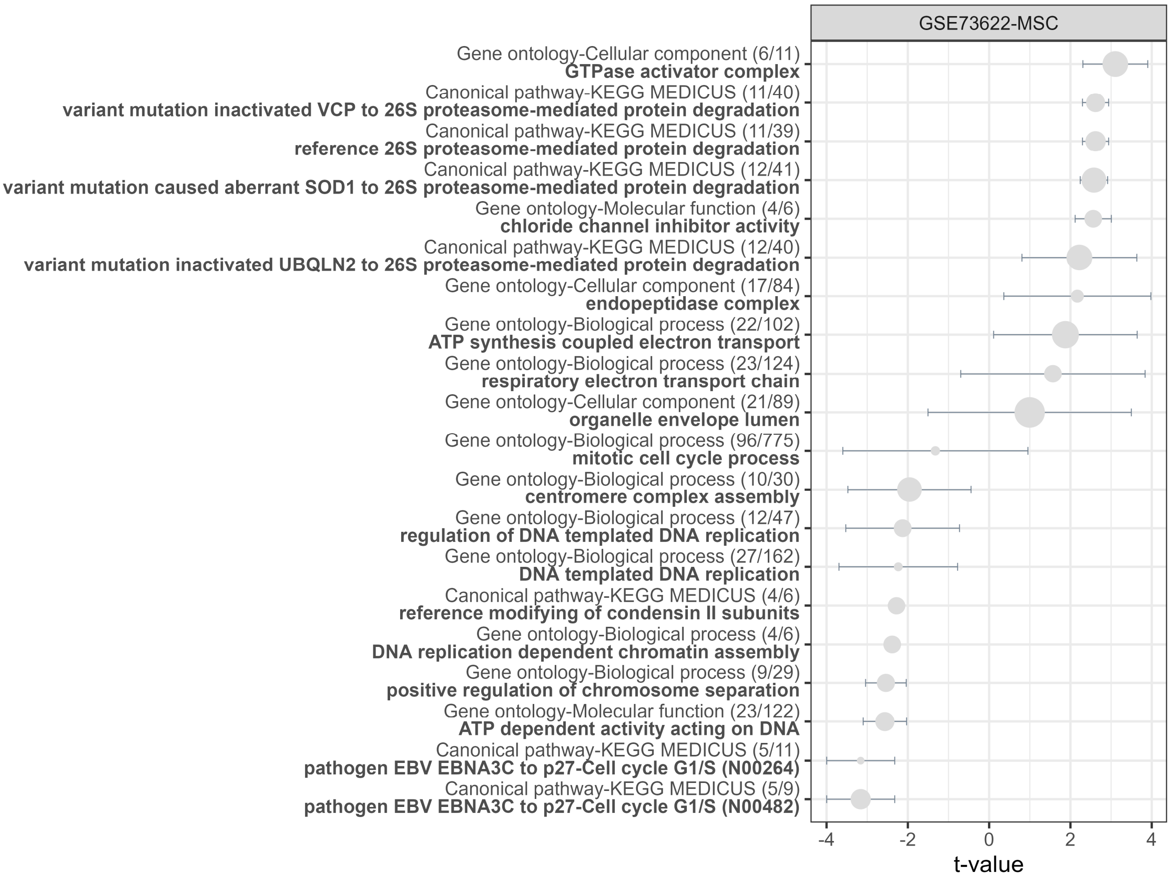

Figures S2. Cell signaling pathways of differentially expressed genes (DEGs) at GSE73622-MSC.

This analysis focuses on the DEGs of cells reprogrammed to the mesenchymal stem cell (MSC) stage from the results included in GSE73622. Graph shows the cell signaling pathways associated with DEGs with *P* below 0.05. The y-axis displays the information about the pathways, with the names of databases containing cell signaling pathways shown at the top. Following this, the total number of genes in each pathway (denoted as the denominator) and the number of DEGs in that pathway (indicated as the numerator) are presented in parentheses. The pathway names are highlighted in bold text. The x-axis represents the t-values of the DEGs within each pathway. Error bars indicate the range of ±1 standard deviation from the mean t-value. Each dot's position corresponds to the mean t-value, while its size reflects the log-transformed *P* from the pathway analysis. The color of the dot represents the significance level of *P*, with gray indicating values equal to or above 0.05. There are no significant pathways.

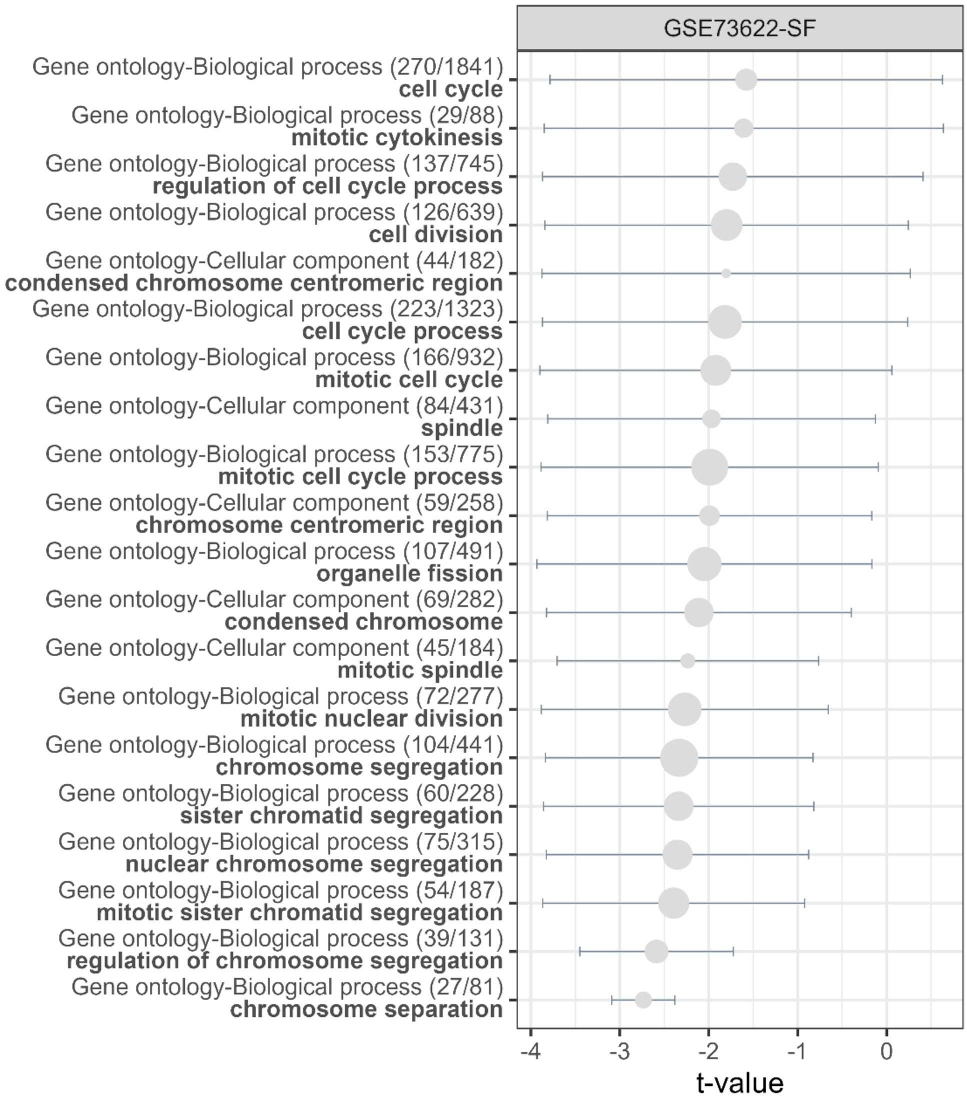

Figures S3. Cell signaling pathways of differentially expressed genes (DEGs) at GSE73622-SF.

This analysis focuses on the DEGs of cells reprogrammed to the stromal fibroblast (SF) stage from the results included in GSE73622. Graph shows the cell signaling pathways associated with DEGs with P below 0.05. The y-axis displays the information about the pathways, with the names of databases containing cell signaling pathways shown at the top. Following this, the total number of genes in each pathway (denoted as the denominator) and the number of DEGs in that pathway (indicated as the numerator) are presented in parentheses. The pathway names are highlighted in bold text. The x-axis represents the t-values of the DEGs within each

pathway. Error bars indicate the range of ±1 standard deviation from the mean t-value. Each dot's position corresponds to the mean t-value, while its size reflects the log-transformed P from the pathway analysis. The color of the dot represents the significance level of P, with gray indicating values equal to or above 0.05. There are no significant pathways.

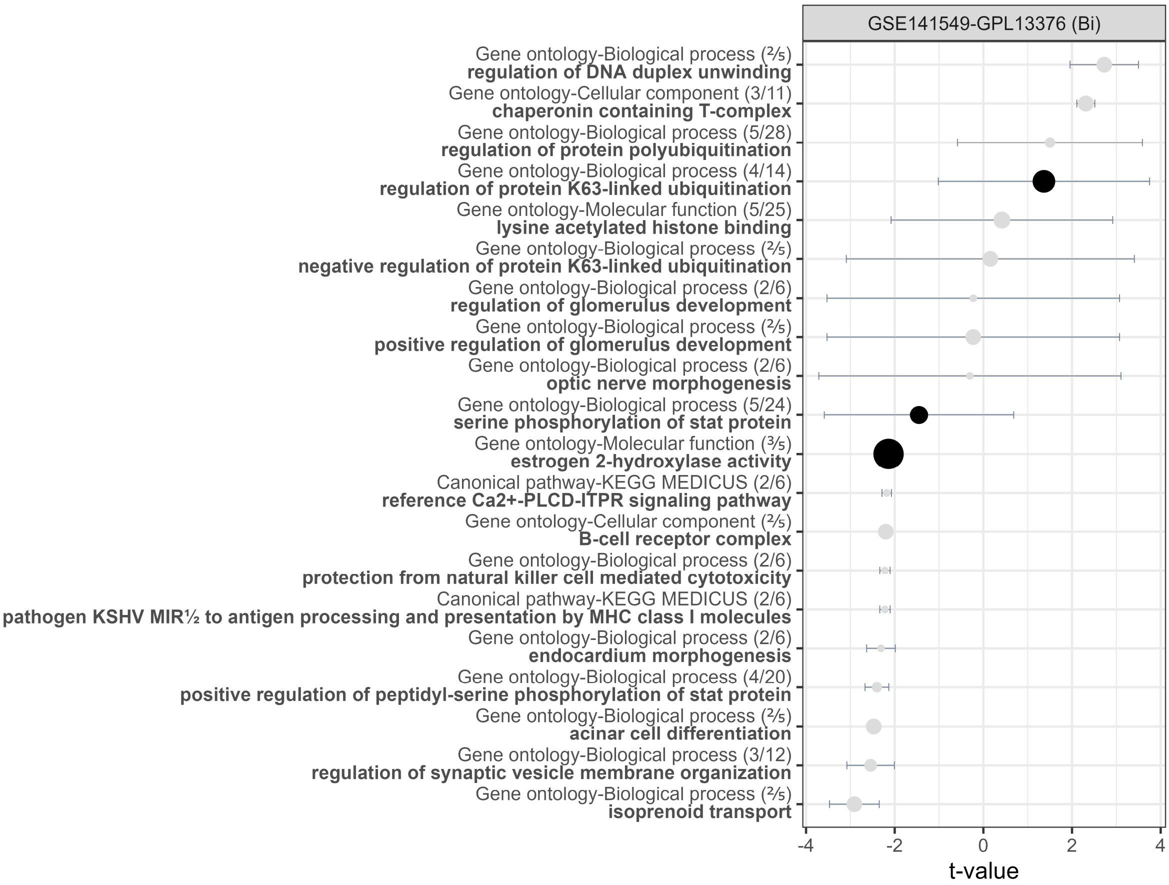

Figures S4. Cell signaling pathways of differentially expressed genes (DEGs) at GSE141549-GPL13376 (Bi).

In GSE141549, samples are analyzed using different platforms. The group in which patient and control samples are analyzed together uses the GPL13376 platform. This analysis utilizes the results of a binomial (Bi) DEG analysis without considering the disease stage of the patients. The analysis identifies the cell signaling pathways associated with DEGs with *P* below 0.05. The y-axis displays the information about the pathways, with the names of databases containing cell signaling pathways shown at the top. Following this, the total number of genes in each

pathway (denoted as the denominator) and the number of DEGs in that pathway (indicated as the numerator) are presented in parentheses. The pathway names are highlighted in bold text. The x-axis represents the t-values of the DEGs within each pathway. Error bars indicate the range of ±1 standard deviation from the mean t-value. Each dot's position corresponds to the mean t-value, while its size reflects the log-transformed *P* from the pathway analysis. The color of the dot represents the significance level of *P*, with black indicating values below 0.05 and gray indicating values equal to or above 0.05.

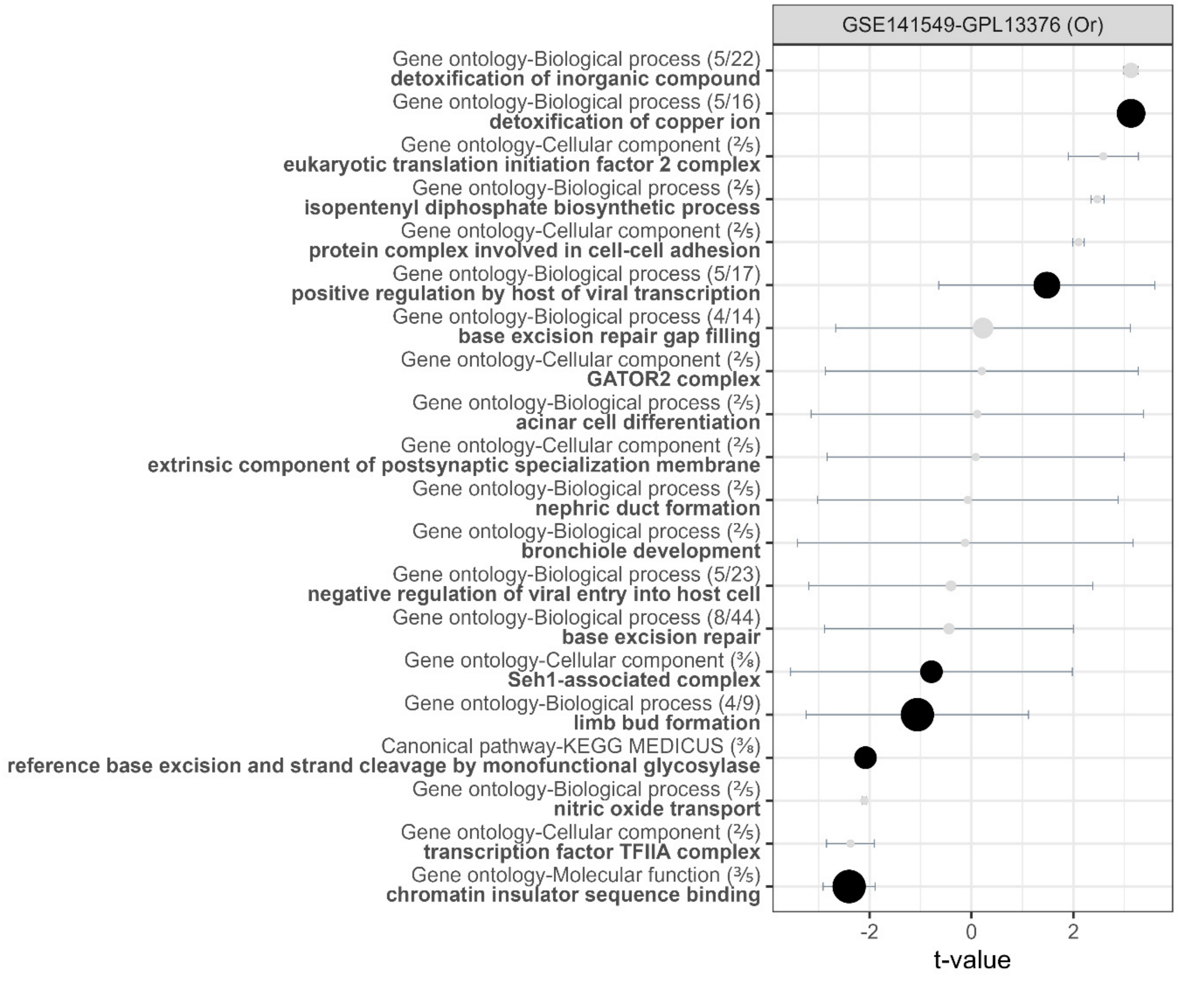

Figures S5. Cell signaling pathways of differentially expressed genes (DEGs) at GSE141549-GPL13376 (Or).

In GSE141549, samples are analyzed using different platforms. The group in which patient and control samples are analyzed together uses the GPL13376 platform. This analysis utilizes the

results of an ordinal (Or) DEG analysis with considering the disease stage of the patients. The analysis identifies the cell signaling pathways associated with DEGs with *P* below 0.05. The y-axis displays the information about the pathways, with the names of databases containing cell signaling pathways shown at the top. Following this, the total number of genes in each pathway (denoted as the denominator) and the number of DEGs in that pathway (indicated as the numerator) are presented in parentheses. The pathway names are highlighted in bold text. The x-axis represents the t-values of the DEGs within each pathway. Error bars indicate the range of ±1 standard deviation from the mean t-value. Each dot's position corresponds to the mean t-value, while its size reflects the log-transformed *P* from the pathway analysis. The color of the dot represents the significance level of *P*, with black indicating values below 0.05 and gray indicating values equal to or above 0.05.

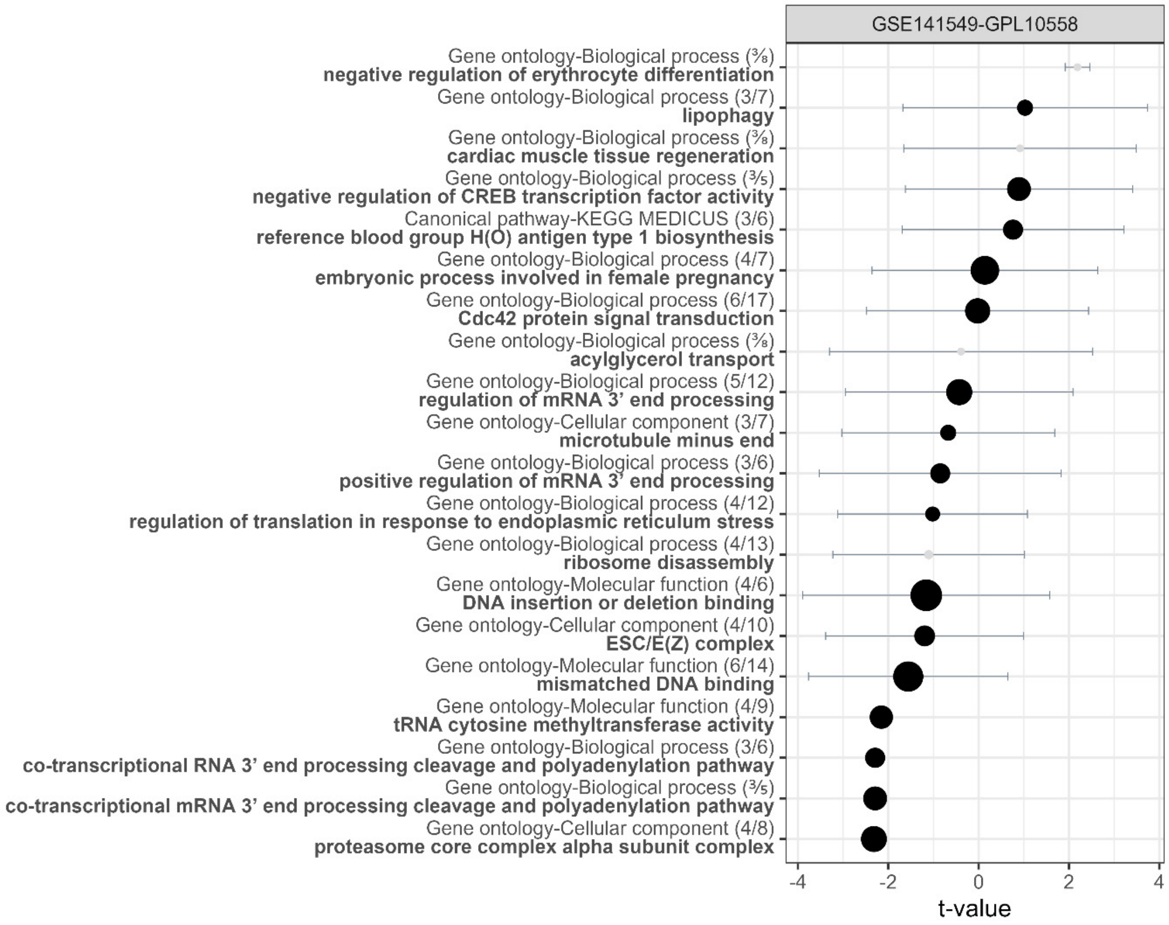

Figures S6. Cell signaling pathways of differentially expressed genes (DEGs) at

GSE141549-GPL10558.

In GSE141549, samples are analyzed using different platforms. The group in which only patient samples are analyzed together uses the GPL10558 platform. This analysis utilizes the results of an ordinal (Or) DEG analysis with considering the disease stage of the patients. The analysis identifies the cell signaling pathways associated with DEGs with *P* below 0.05. The y-axis displays the information about the pathways, with the names of databases containing cell signaling pathways shown at the top. Following this, the total number of genes in each pathway (denoted as the denominator) and the number of DEGs in that pathway (indicated as the numerator) are presented in parentheses. The pathway names are highlighted in bold text. The x-axis represents the t-values of the DEGs within each pathway. Error bars indicate the range of ±1 standard deviation from the mean t-value. Each dot's position corresponds to the mean t-value, while its size reflects the log-transformed *P* from the pathway analysis. The color of the dot represents the significance level of *P*, with black indicating values below 0.05 and gray indicating values equal to or above 0.05.

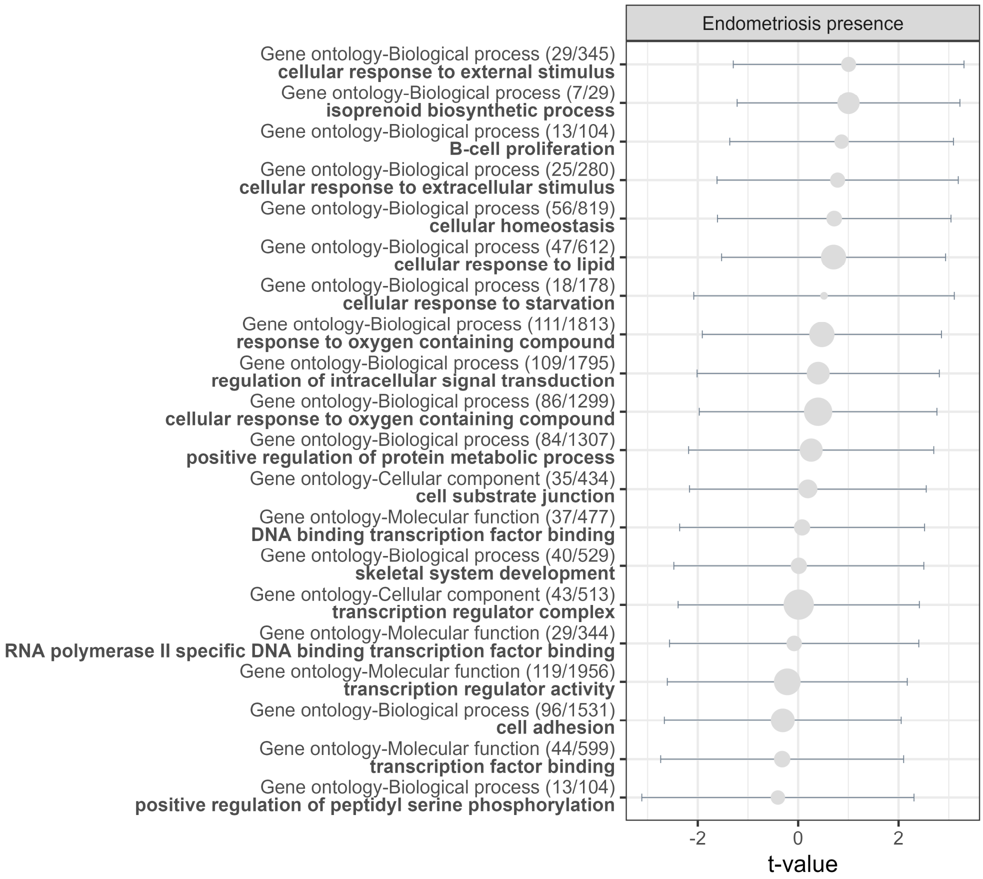

Figures S7. Cell signaling pathways of differentially expressed genes (DEGs) at endometriosis presence meta-analysis.

The analysis identifies the cell signaling pathways associated with DEGs with *P* below 0.05. The y-axis displays the information about the pathways, with the names of databases containing cell signaling pathways shown at the top. Following this, the total number of genes in each pathway (denoted as the denominator) and the number of DEGs in that pathway (indicated as the numerator) are presented in parentheses. The pathway names are highlighted in bold text. The x-axis represents the t-values of the DEGs within each pathway. Error bars indicate the range of ±1 standard deviation from the mean t-value. Each dot's position corresponds to the mean t-value, while its size reflects the log-transformed *P* from the pathway analysis. The color of the dot represents the significance level of *P*, with gray indicating values equal to or above 0.05. There are no significant pathways.

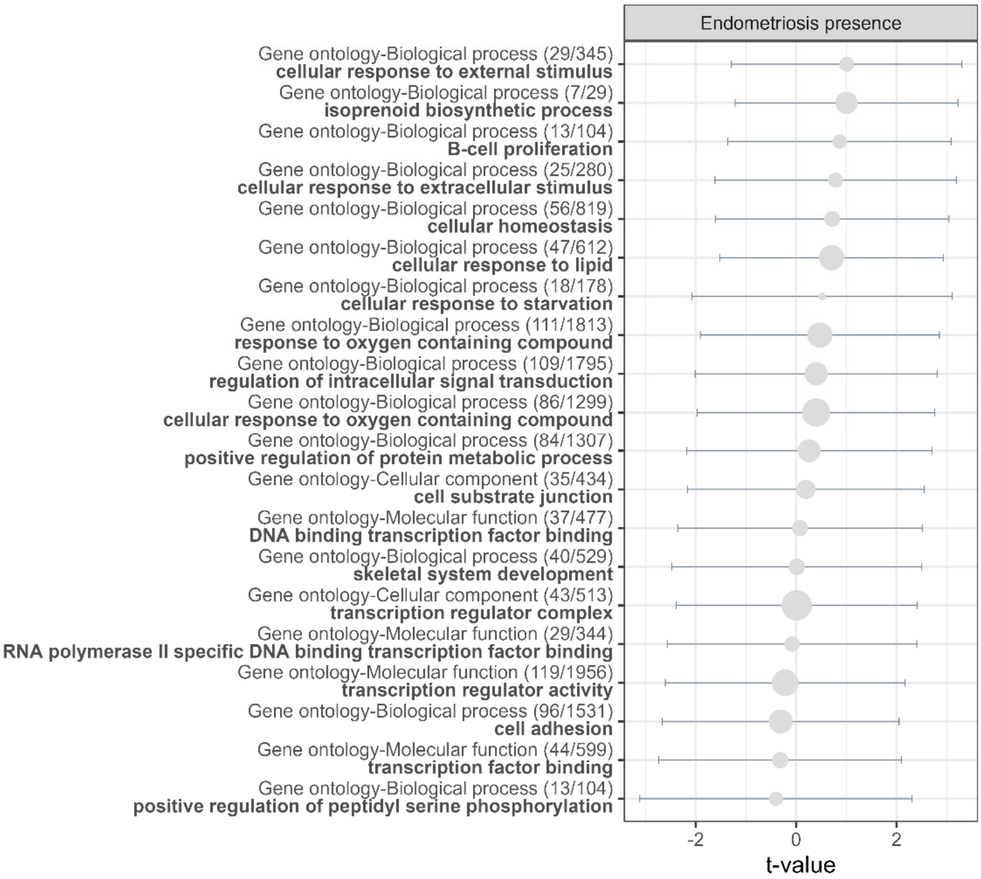

Figures S8. Cell signaling pathways of differentially expressed genes (DEGs) at endometriosis presence meta-analysis.

The analysis identifies the cell signaling pathways associated with DEGs with *P* below 0.05. The y-axis displays the information about the pathways, with the names of databases containing cell signaling pathways shown at the top. Following this, the total number of genes in each pathway (denoted as the denominator) and the number of DEGs in that pathway (indicated as the numerator) are presented in parentheses. The pathway names are highlighted in bold text. The x-axis represents the t-values of the DEGs within each pathway. Error bars indicate the range of ±1 standard deviation from the mean t-value. Each dot's position corresponds to the mean t-value, while its size reflects the log-transformed *P* from the pathway analysis. The color of the dot represents the significance level of *P*, with gray indicating values equal to or above 0.05. There are no significant pathways.
